# A commentary on ‘Machine learning based peri-surgical risk calculator for abdominal related emergency general surgery: a multicenter retrospective study’

**DOI:** 10.1097/JS9.0000000000001434

**Published:** 2024-04-09

**Authors:** Tianyang Mao, Xin Zhao, Qingyun Xie, Fengwei Gao

**Affiliations:** a North Sichuan Medical College, Nanchong; b Department of Hepato-Pancreato-Biliary Surgery, The People’s Hospital of Leshan, Leshan; c Liver Transplantation Center, State Key Laboratory of Biotherapy and Cancer Center, West China Hospital, Sichuan University and Collaborative Innovation Center of Biotherapy, Chengdu, People’s Republic of China

*Dear Editor,*

I have read an interest article entitled ‘Machine learning based peri-surgical risk calculator for abdominal related emergency general surgery: a multicenter retrospective study’, published in the International Journal of surgery^1^. The study employed five machine learning algorithms to construct predictive models for postoperative complications in emergency general surgery patients. Additionally, the study designed a web-based risk prediction calculator. The models were used to predict postoperative mortality, pneumonia, surgical site infection, thrombosis, and prolonged mechanical ventilation (>48 h), which was crucial for guiding physicians in formulating treatment strategies. The results indicated that random forest model was the best, which increased the reliability and robustness of the predictive model. However, the following certain aspects require further clarification.

Firstly, while this study focused on predicting perioperative risk events, the model construction was limited to preoperative data. Intraoperative events also played a crucial role in mortality and complications. Factors such as the type of surgery, extended operation duration, intraoperative hypotension, etc., can lead to an increase in postoperative complications occurrences^2,3^. Simultaneously, the classification of hospitals into four grades suggested potential variations in the expertise level of operating doctors, which could affect the study results. The exclusion of the above data might introduce bias to the accuracy of the results, potentially underestimating the occurrence of risk events in certain patients, posing significant risks.

Secondly, this study focused on eight common acute abdominal diseases such as acute appendicitis, acute cholecystitis, hernia, and acute intestinal obstruction. The study of Lluis *et al*.^4^ showed that the incidence of postoperative complications was different across different acute abdominal diseases. However, the author omitted disease classification data into the model construction. Meanwhile, I attempted to use the interactive questionnaire system of this study. It was observed that the incidence of postoperative risk events remained constant in the same patient with different diseases and different computed tomography scans, presenting an inconsistency with the actual situation.

Thirdly, this study enrolled patients from five medical centers for research purposes. With 7695 patients in the derivation cohort, belonging to a large sample size study, which was conducive to improve the model accuracy. However, the lack of external validation raised uncertainties regarding the generalizability to other medical institutions in China or even Asia. It is recommended to incorporate external data to further verify the applicability of this model.

Overall, the authors’ work merit acknowledgment, and this study can provide valuable guidance for the field of emergency general surgery. I believe that if the authors could address these issues or provide clarifications, it would significantly elevate the relevance and impact of the study.

## Ethical approval

Not applicable.

## Consent

Not applicable.

## Sources of funding

This research did not receive any specific grant from funding agencies in the public, commercial, or not-for-profit sectors.

## Author contribution

T.Y.M. : study design and writing; X.Z. and Q.Y.X.: make suggestions; F.W.G.: study supervision.

## Conflicts of interest disclosure

No conflicting relationship exists for any of the authors.

## Research registration unique identifying number (UIN)

1. Name of the registry: not applicable.
2. Unique identifying number or registration ID: not applicable.
3. Hyperlink to your specific registration (must be publicly accessible and will be checked): not applicable.

## Guarantor

Pro. Fengwei Gao, Liver Transplantation Center, No. 37, Guoxue Lane, Wuhou District, Chengdu, 610041, Sichuan Province, People’s Republic of China. E-mail: gaofengweigfw8@126.com.

## Date availability statement

All data generated or analyzed during this study are included in this article. The data are available from the corresponding author upon reasonable request.
